# The essential conditions needed to implement the Indigenous Youth Mentorship Program: a focused ethnography

**DOI:** 10.1186/s12889-021-12412-1

**Published:** 2022-02-02

**Authors:** Frances Sobierajski, Lucie Lévesque, Jonathan McGavock, Tamara Beardy, Genevieve Montemurro, the IYMP National Team, Frances Sobierajski, Lucie Lévesque, Jonathan McGavock, Tamara Beardy, Kate Storey, Kate Storey

**Affiliations:** 1 School of Public Health, University of Alberta, Edmonton Clinic Health Academy, 11405 – 87 Avenue, Edmonton, Alberta T6G 1C9 Canada; 2 School of Kinesiology and Health Studies, Queen’s University, 28 Division St, Kingston, Ontario Canada; 3 Department of Pediatrics and Child Health, Faculty of Health Sciences, University of Manitoba, Children’s Hospital Research Institute of Manitoba, 715 McDermot Ave, Winnipeg, Manitoba Canada

**Keywords:** Comprehensive school health, Essential conditions, Indigenous health, Implementation science, Health promotion, Peer mentorship, Qualitative research

## Abstract

**Background:**

The Indigenous Youth Mentorship Program (IYMP) is a 20-week communal, relationship-based afterschool healthy living program for Indigenous youth in Canada. IYMP embraces the Anishnaabe/Nehiyawak concepts of Mino-Bimaadiziwin/miyo-pimâtisiwin (“living in a good way”) via its core components of physical activities/games, healthy snacks, and relationship-building. A strength of IYMP is that it values autonomy, adaptability, and the school community context. However, this presents challenges when evaluating its implementation, given that traditional implementation science methods tend to oversimplify the process. In response, essential conditions for the implementation of school-based healthy living programs across diverse contexts have been developed. The purpose of this research was to understand the applicability of these essential conditions within the context of IYMP.

**Methods:**

15 participants (*n* = 10 Young Adult Health Leaders; *n* = 5 coordinators) with experience implementing IYMP in the provinces of Alberta, Saskatchewan, Manitoba, and Quebec were purposefully sampled. Focused ethnography was the guiding method and one-on-one semi-structured interviews were used as the data generation strategy. The purpose of the interviews was to understand what conditions are needed to implement IYMP. The interview guide was based on previously established essential conditions developed by the research team. Interviews were audio-recorded and transcribed, and content analysis was used to identify patterns in the data.

**Results:**

The overarching theme that emerged from the interviews was the applicability of the essential conditions when implementing IYMP. Participants felt the eight core conditions (*students as change agents*, *school/community-specific autonomy*, *demonstrated administrative leadership*, *higher-level support*, *dedicated champion(s) to engage school community*, *community support*, *quality and use of evidence*, and *professional development*) and four contextual conditions (*time*, *funding and project support*, *readiness and understanding*, and *prior community connectivity*) were necessary, but made suggestions to modify two conditions (*youth led* and *learning opportunities*) to better reflect their experiences implementing IYMP. In addition, a new core condition, *rooted in relationship*, emerged as necessary for implementation.

**Conclusions:**

This research adds to the literature by identifying and describing what is needed in practice to implement a communal, relationship-based afterschool healthy living program. The essential conditions may support other researchers and communities interested in implementing and rippling similar programs.

**Supplementary Information:**

The online version contains supplementary material available at 10.1186/s12889-021-12412-1.

## Background

Hundreds of healthy living interventions for youth are implemented each year, however, few of these are tailored to the needs of Indigenous youth [1, 2]. Among these interventions, a smaller number ripple (our preferred word for scale-up) to multiple sites, and little information exists describing this process. To date, only five interventions have rippled out to multiple Indigenous communities in Canada [3–7]. The limited research in this area revealed that school-based, peer mentorship programs are effective for promoting health and well-being [4, 7]. An important feature of these effective programs is the use of a comprehensive school health (CSH) approach. CSH applies a socioecological framework to create a healthy school culture, promote healthy lifestyle behaviours, and enhance student educational outcomes [8]. Despite having established effectiveness, more information is needed to understand how CSH interventions can be rippled to diverse Indigenous school communities. Communities, researchers, practitioners, policymakers, funders, and other partners are looking for guidance, and this research will support implementation, rippling, and sustainability [9].

Collecting information throughout implementation can reveal how and why interventions were successfully taken up and maintained by communities in diverse settings, which can guide future rippling [10]. Two landmark healthy living interventions for Indigenous youth, The Sandy Lake Health and Diabetes Prevention Project and Kahnawà:ke Schools Diabetes Prevention Project, provide foundational knowledge for implementing healthy living interventions for Indigenous youth in community settings. Research from these projects identified key implementation factors, including building on pre-existing relationships, facilitating the sharing of information, and prioritizing Indigenous voices and leadership [11]. Identifying commonalities within the intervention context across these two programs provided evidence of ‘common key community characteristics’ to support their long-term maintenance. However, this information pertained to the delivery of these interventions in a single community. As these programs did not study the rippling of their interventions into new Indigenous school community settings, the common features needed to successfully ripple a communal, relationship-based healthy living program to new Indigenous school communities remains unclear.

Shortcomings in implementation research may be due, at least in part, to traditional implementation science outcomes (i.e., reach, recruitment, dose, and fidelity) that tend to oversimplify the implementation process. In response to this, implementation scientists are increasingly utilizing qualitative methods to unpack the how and why behind program implementation [10]. For example, a secondary analysis of qualitative data exploring the process of implementing school-based healthy living programs using a CSH approach was undertaken and identified a set of essential conditions necessary for implementation [12, 13]. The essential conditions included both core conditions (i.e., *students as change agents*, *school/community-specific autonomy*, *demonstrated administrative leadership*, *higher-level support*, *dedicated champion(s) to engage school community*, *community support*, *quality and use of evidence*, and *professional development*) and contextual conditions (i.e., *time*, *funding and project support*, *readiness and understanding*, and *prior community connectivity*). Core conditions are those that are necessary for implementation whereas contextual conditions determine whether or not the core conditions can be achieved. These conditions have been adapted by schools across Canada and may be applicable to other school-based healthy living programs because of their focus on commonalities within the implementation process rather than commonalities within the intervention [13]. As such, they provide school communities with important information about the core conditions that are needed to create a school culture that promotes healthy living without prescribing the specific types of activities required. These conditions allow stakeholders to plan, develop, and implement intervention activities that are autonomous and fit the unique context and needs of the community. Thus, these previously established essential conditions may provide insight on how to implement and ripple school-based healthy living programs for Indigenous youth while promoting autonomy, adaptability, and context. However, these conditions have yet to be applied in the context of school-based, peer mentorship programs being delivered within Indigenous school communities.

Therefore, the goals of this study were two-fold: 1. to understand whether the essential conditions resonated with participants’ experience of implementing a communal, relationship-based afterschool healthy living program, The Indigenous Youth Mentorship Program (IYMP) and 2. how the essential conditions needed be modified to reflect the experience of implementing IYMP. Understanding participants’ experience of implementing IYMP across diverse contexts is important to inform the implementation and rippling of school-based, healthy living programs for Indigenous youth.

### Intervention: the Indigenous Youth Mentorship Program (IYMP)

IYMP is a communal, relationship-based peer-led afterschool healthy living program [4, 14–20]. IYMP is delivered by Indigenous high school students for elementary school students with the support of a Young Adult Health Leader (YAHL) chosen by the community. IYMP promotes wholistic wellness, healthy behaviours aimed at preventing type 2 diabetes, and Mino-Bimaadiziwin/miyo-pimâtisiwin (the way of the good life in Anishinaabe/Woodland Cree). The focus on Mino-Bimaadiziwin/miyo-pimâtisiwin was a priority for Elders and Knowledge Keepers on our team as this reflects an Indigenous worldview of wholistic health and wellness [21]. IYMP is guided by the teachings of Indigenous scholars Drs. Martin Brokenleg and Verna Kirkness and is centered on Indigenous philosophies and culturally affirming educational approaches, the Circle of Courage [4, 22, 23] and the Four Rs: Respect, Relevance, Reciprocity, Responsibility [4, 24]. For additional information about IYMP’s theoretical framework see Appendix 1. The core components of IYMP include: 1) physical activity/games, 2) healthy snacks, and 3) relationship building. IYMP is usually offered once per week for 90 min from January to June or sometimes throughout the entire school year. IYMP is relationship-driven and builds on the strengths, energy, and talents of youth and communities. Each week, high school mentors meet as a group to plan each 90-min session. During the program, mentors are responsible for setting up the activity areas, running the activities, and cleaning up. Mentors also prepare and serve the healthy snack. High school mentors are encouraged to tailor activities to meet the needs of their community while still embodying the core components of IYMP. YAHLs in each community support mentors as they plan and deliver the program. IYMP can be delivered in diverse settings, including elementary schools, high schools, or local community centers.

YAHLs receive support from regional program coordinators who provide assistance with training, implementation, and evaluation. There is also a wider Canadian network of youth, community leaders, Elders, scientists, and knowledge users from over 30 communities and five universities to support implementation. Regional and national gatherings are hosted annually. During the gatherings, IYMP team members receive Indigenous teachings of wholistic health from Elders and knowledge keepers, participate in land-based activities, reflect on Mino-Bimaadiziwin/miyo-pimâtisiwin, and share their experience delivering IYMP.

IYMP started in 2004 as a community-based, research project designed to build on the leadership skills of Indigenous high school students in northern Winnipeg [4, 14–17]. In 2010, the urban mentor model was adapted for delivery in a remote Anishininiimowin (Oji-Cree) community in Northern Manitoba (Kistiganwaacheeng First Nation) [4, 16]. In this remote and isolated Indigenous community, IYMP prevented weight gain and reduced risk factors for type 2 diabetes in Indigenous children [4]. Follow-up studies revealed the effect size remained significant when rippled to two and then five communities (In preparation). The term rippling was chosen over the Western term 'scaling up' by Elders, Knowledge Keepers, and leaders from Indigenous communities involved in IYMP. The term rippling aligns with IYMP’s guiding principles and Indigenous governance models in which voices from the community are prioritized and honoured including Indigenous ways of knowing that convey knowledge through "personal stories, wholistic perspectives and metaphoric language" [25]. Rippling was also considered a more natural description of the team’s approach to engaging new communities into the program.

IYMP has received the MacJannet Prize (2014) for community development and the Manitoba Mino Bimaadiziwin Award (2015) for promoting living in a good way. It is a recognized ‘best practice’ on the Public Health Agency of Canada’s Best Practices Portal: Aboriginal Ways Tried and True [26]. As such, there is convincing evidence that peer-led approaches are effective for improving health outcomes among Indigenous youth. Most recently, the program has rippled across Canada, and thousands of children and youth now participate across five different provinces [19, 20]. IYMP communities are diverse in geography, size, background and Indigenous cultures. Therefore, IYMP provides a unique opportunity to explore what conditions across communities are needed for implementation.

## Method

This research was approached from an Indigenist, anti-colonial framework, which outlines the process of doing research in a good way across Indigenous and Settler contexts [27]. At its core, Indigenous approaches to health promotion prioritize self-determination, community voices and priorities, and wholistic views of wellness. It acknowledges the lasting effects of colonization, including residential schools, in creating and perpetuating health inequities faced by Indigenous peoples and strives to counteract this oppression. By centering the research around trusting relationships and Indigenous knowledge, it is possible to create a space for deep learning.

This study employed qualitative methods, and aligned with an Indigenist, anti-colonial framework, whereby knowledge was produced through interactions between participants and researchers to create a space for mutual understanding [27, 28]. The questions guiding this study were: do the essential conditions resonate and if so, how can they be modified to reflect participants’ experience implementing IYMP? Focused ethnography was used as the method to address these questions, as it allowed us to investigate the shared experience of implementing IYMP in specific contexts [29, 30]. This method also aligned with the anticipated outcome of the study to provide relevant and meaningful information to inform and improve the implementation of IYMP.

This study was developed and supported by IYMP community members, provincial organizations, and researchers. Collectively, the team had a depth of knowledge and expertise within the field of health promotion, implementation science, and Indigenous health. Indigenous methods of interpretation were prioritized in alignment with IYMP’s guiding principles and our team’s Indigenist, anti-colonial approach. As lead author, FS acknowledges the need to situate herself within the context of this study. FS is a non-Indigenous, female graduate student with a background in health promotion and medicine. Through her studies, FS was passionate about supporting the health and wellbeing of children and youth and developed an understanding how to conduct research in a good way to honor and support Indigenous youth. Communities voiced that programs were looking for guidance on how best to facilitate programming. In collaboration with the wider IYMP network, the present study was designed. The goal of this research was to prioritize community voice and create positive change by developing relevant materials to support communities as they plan and deliver IYMP programming. This manuscript is in accordance with standards for reporting qualitative research [31].

### Participant recruitment

YAHLs and program coordinators were purposefully sampled because they had experience running and/or coordinating IYMP [29]. YAHLs were directly involved in running the program in their community and coordinators supported several communities. Thus, participants could provide a rich understanding of what was needed for implementation. Participants were identified and recruited through our IYMP network. First, the lead author invited coordinators (*n* = 6) to participate in the study in person, by phone, or by email. If participants expressed interest in participating, they received an information letter. Upon agreement to participate, an interview was scheduled. Before starting the interview, participants provided written and verbal informed consent. Next, we asked coordinators to identify and invite YAHLs who had at least 1 year of experience running the program (*n* = 12) to participate in the study. Participants who had less than 1 year of experience were not eligible because they would not have had the experience or context working with IYMP to provide in-depth responses, since program implementation is a year-long process. If YAHLs expressed interest in participating, the lead author approached participants with an information letter and sought written and verbal consent prior to the interview. Target recruitment of 10–20 participants was selected based on previous research utilizing qualitative methods to investigate the implementation of healthy living programs for Indigenous youth [3, 5].

### Participants

Ten YAHLs and five coordinators participated. YAHLs and coordinators delivered IYMP in their school communities for one to 4 years. Nine identified as female and six as male. YAHLs included individuals who were university student volunteers (*n* = 3), teachers (*n* = 2), youth center staff (*n* = 2), educational assistants (*n* = 2), and support staff (*n* = 1). As such, most YAHLs (*n* = 7) worked in the community prior to becoming involved with IYMP. Coordinators were hired staff. Altogether, the 15 participants represented communities from Alberta, Saskatchewan, Manitoba, and Quebec.

### Data generation

All data generation strategies and approaches were co-designed and developed with the IYMP team, which honoured our team’s guiding principles. A semi-structured interview guide was co-developed and approved by communities and members of our National Advisory Circle, and one-on-one interviews were conducted with YAHLs and coordinators. Interviews were conducted by the lead author and ranged in duration from 20 to 90 min (average = 47 min; standard deviation = 16 min). When possible, interviews were conducted in-person (*n* = 8). This decision was made based on feedback from the IYMP team that face-to-face interviews were essential to develop positive relationships and open dialogue. Thus, when feasible, the lead author travelled by car and/or plane to meet the participants. However, in some cases (*n* = 7) it was not possible to conduct the interviews in-person, so phone interviews were scheduled to ensure participation regardless of geographic location.

The goal of the interview was to draw on the experience and knowledge of YAHLs and coordinators to understand what is needed to implement IYMP. Interview guide development was based on the previously established essential conditions described above in collaboration with communities [12, 13]. More specifically, the interview guide was structured to include introductory, main, and summary questions. Introductory questions were open-ended and allowed participants to share how they became involved in IYMP and their experience implementing IYMP. The main questions explored whether the essential conditions resonated with their experiences’ of IYMP. Participants were asked if the essential conditions were necessary to implement IYMP (e.g., do you feel this condition is needed), and if the essential conditions were a part of their IYMP experience (e.g., do you feel this condition is present in your community or the communities you work with). Participants were also asked if any other conditions needed to be added, or if modifications were required. Summary questions were used to wrap-up the conversation and provide participants with an opportunity to share any other ideas. The interview guide was structured to blend open-ended and structured questioning to honor participants narrative while also building on the previously established essential conditions. Throughout the interview, the lead author created a space for participants to share their story and actively listened.

Participant observation and field notes were also used as a data source [29]. These notes captured the lead author’s general impressions and main findings from each interview. The lead author also engaged in participant observation and field note writing while working alongside IYMP research coordinators. Within the context of this study, participant observation and field note writing was an active process. The lead author spent time in community building relationships, receiving teachings from Elders and knowledge keepers, and engaging in ceremony. Some of her most meaningful experiences were learning by actively participating in IYMP activities alongside youth. Her reflections of these experiences helped her develop an understanding of what it looked like in action to support communities as they implemented IYMP. Most importantly, these experiences and teachings provided a connection to the concept of Mino-Bimaadiziwin/miyo-pimâtisiwin (“living in a good way”) not just in theory, but how it is lived. These were invaluable learnings that could not have been captured through interviewing alone.

### Data analysis/synthesis

In alignment with our Indigenist, anti-colonial approach, we use the term ‘synthesis’ alongside ‘analysis’ to indicate that data are brought together versus torn apart. Following data generation, interviews were were audio recorded and transcribed verbatim yielding 488.5 single-spaced pages. Participants were engaged to interpret the findings, reflect on their meaning, and consider how the data were re-presented. Transcripts were reviewed and organized using the Atlas.ti 8 software package. Content analysis was used as it is considered the most appropriate analytic strategy for focused ethnography [29, 30]. Based on an established protocol to explore the applicability of the essential conditions in other settings [13], the first step of the analysis was to read each transcript line-by-line to deductively identify and code segments of data broadly referring to the essential conditions [29, 32]. The use of a deductive approach helped organize the data around the essential conditions. Next, data grouped by condition were inductively coded. This step explored whether participants indicated that the condition was necessary. Patterns in the data were identified and refined in order to operationalize how each condition needed to be adapted to align with participants’ experience of implementing IYMP. The inductive approach also enabled the development of a new condition. Taken together, the use of deductive and inductive approaches allowed the analysis to build on previous knowledge while remaining open to new learnings. Field notes were incorporated to understand how the essential conditions could be applied in practice. The lead author also engaged in memoing at all stages to capture emerging theoretical notions. As part of the analysis and synthesis of results, illustrative quotes were selected to support study findings. Based on feedback from the IYMP team (including participants), duplicated words and words such as “um, ahs, like and yeah” within quotes were removed (e.g., “conversation guide that I um, that I made” became “conversation guide that I made”). This decision aligns with recommendations from Standing [33]. Quotes were also de-identified to maintain the confidentiality of participants and communities.

### Methodological rigour

To achieve trustworthiness throughout the research process, strategies were employed to establish credibility, transferability, dependability and confirmability [34]. First, strategies used to enhance credibility included in-depth interviews, prolonged engagement working alongside IYMP research coordinators, and visiting IYMP programs to become more familiar with how they run in each context. The second criteria, transferability, was enhanced through participant recuitment from communities that are diverse in geography, size, culture and governance. Finally, dependability and confirmability were achieved by attending to methodological congruence, revisiting research findings, documenting decisions, and debriefing with colleagues.

Most importantly, the lead author acknowledges that the study findings are a re-presentation of participants’ experiences based on interpretations of the data. Throughout the research process, the lead author took care to actively listen and synthesize participants’ experiences. Emerging findings were then shared back with all partners to ensure findings prioritized and honored communities’ voice.

### Research ethics

This project received ethics approval through the Human Research Ethics Board at the University of Alberta (Pro000695330) and is in accordance with the standards set by Ownership, Control, Access and Possession (OCAP)®. It was performed in accordance with relevant guidelines and regulations. Data are owned and controlled by the communities involved, and all findings were approved by the IYMP National Advisory Circle prior to publication.

In alignment with our Indigenist, anti-colonial approach [28], true guidance of IYMP comes from communities and our model of Kanien’kehá:ka decision making [35]. As such, this project was guided by our National Advisory Circle and Indigenous models of governance [36–38]. The National Advisory Circle is made up of Elders, community leads, and researchers. The National Advisory Circle provided guidance at all stages of the research process. This included co-developing the data generation strategy and interview guide, reviewing and contextualizing research findings, and identifying strategies to share research findings.

## Results

The overarching theme that was generated from the interviews with YAHLs and coordinators was the applicability of the essential conditions to their experience implementing IYMP. During the interviews, participants seemed comfortable sharing their perspectives regarding the essential conditions. Indeed, when participants agreed or disagreed with the applicability of a given condition, they provided rich descriptions of why it was necessary or how it needed to be adapted to fit the IYMP context. For example, at the onset of interviews, a few YAHLs and coordinators were apprehensive about how the conditions could apply in diverse contexts. However, the YAHLs and coordinators were surprised after the essential conditions were presented back to them, as the conditions resonated with their school community and fit within the larger IYMP context. Some words used to describe the applicability of the essential conditions to the IYMP context included: *“Absolutely” (P3)*, *“Yes definitely” (P12)*, and *“Very essential” (P1).* Participants also provided descriptions of what the conditions looked like in action within the context of IYMP. In fact, in one province, the essential conditions were already being used in this manner. During an interview, a coordinator explained how they used the essential conditions to guide their conversations with new communities. They described their approach to working with a new community as:

I would talk to [a new community] about these are the different ways I’ve seen it run. I have a conversation guide that I made, framed around these essential conditions, to go through each one and kind of talk about what will you, what will work in your school (P6).

**Fig. 1:**
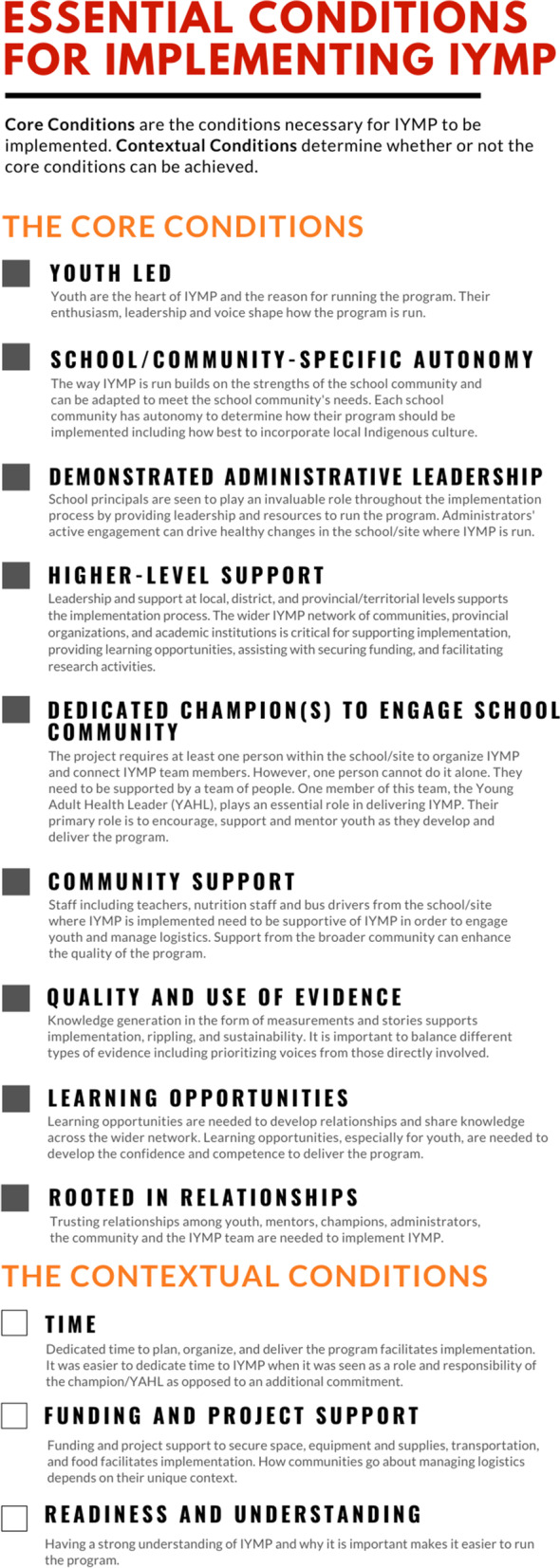
Essentials conditions for implementing IYMP

More specifically, participants agreed that the eight core conditions were necessary to implement IYMP but had suggestions for how two of the conditions could be adapted to fit the context of IYMP. Additionally, a new core condition, *rooted in relationships,* emerged from the analysis as necessary for implementation and replaced the condition *prior community connectivity* (see Table 1 for a summary of the essential conditions and their modifications). A detailed description of each condition is presented below with supporting quotes and resulting adaptations. A summary figure is also included to illustrate the core and contextual conditions for implementing IYMP (see Fig. 1).

**Table 1:** Summary of modifications to the essential conditions to fit the IYMP context

Original	IYMP Modifications
CORE CONDITIONS	
Students as change agents	Youth led
School/community-specific autonomy	- No change
Demonstrated administrative leadership	- No change
Higher-level support	- No change
Dedicated champion(s) to engage school community	- No change
Community support	- No change
Quality and use of evidence	- No change
Professional development	Learning opportunities
	Rooted in Relationships
CONTEXTUAL CONDITIONS	
Time	- No change
Funding and project support	- No change
Readiness and understanding	- No change
Prior community connectivity	Became the core condition “Rooted in Relationships”

### Core conditions

#### Youth led

All participants agreed that students are the heart of IYMP – and are the reason communities run the program. YAHLs and coordinators wanted to be involved in IYMP because it prioritizes mentorship, empowers youth, and promotes healthy living. They also agreed that youth enthusiasm, leadership, and voice shaped how the program was run. As one participant stated: *“[it’s] the main goal underneath everything” (P11).* Indeed, youth engagement was identified as an indicator of successful implementation*.* One participant noted:

If you can get a group of kids enthusiastic about it, and wanting to participate, and really just emphasize that the program is about showing their strengths and their knowledge, and doing what they want to do. That’s probably the biggest thing to run the program successfully is just getting youth engaged (P10).

Similarly, youth leadership was identified as essential, and participants emphasized how youth serve as active leaders in IYMP. As one participant commented: *“the students are the active leaders and planners in the program, especially those high school mentors” (P7).* Another participant explained: *“for me [the] standout would be the mentors, and their ability make decisions, and decide things” (P2).* Participants expressed how youth leadership was especially important within the context of a peer mentorship program. As expressed by one participant: *“if the goal is to bring grade four kids into a program, that mentorship program, then you want to surround them with the high [school] students who are – who would be good role models” (P11).* Notably, participants prioritized voices from youth when planning and making changes to the program. One participant described engaging with youth in the program to learn: *“how do they want to see the program”* (P1). They believed that this approach enhanced engagement and passion for the program. Therefore, this condition was adapted to *youth led* to reflect the role of youth enthusiasm, leadership, and voice in shaping how the program is run.

#### School/community-specific autonomy

All participants agreed that the delivery of IYMP must build on communities’ strengths and needs to be adaptable to the unique community context. Participants felt that it would not have been possible to run the program without autonomy. For example, in some communities, IYMP was adapted to run during school hours instead of afterschool. One participant explained how this modification *“was kinda required” (P10)* because it meant that teachers could provide supervision during the IYMP program and youth could participate without needing to arrange for transportation after the program ended. Overall, it was described that IYMP is not a one-size-fits-all approach. As one participant explained:

I think that’s what makes it appealing to communities that it can be so adaptive to their unique context because two communities right next door to each other will have totally different needs and parent involvement, and availability of resources, and equipment. So, I think the more that we can adapt it the more it can be taken up by other communities and actually be sustainable (P6).

Participants also explained how *school/community-specific autonomy* promoted program relevance because any changes to how the program was implemented came from those directly involved based on their understanding of the school/community’s strengths and needs. As one participant indicated: “*I also think just like working with the teachers and principals, and getting – being able to work with them to figure out what worked best, was super important, otherwise I just really think the program would not have happened here” (P7).* This is echoed by another participant who stated:

[community members] know your community better than anyone else does, and you know your kids better than anyone else does; ultimately this is about you guys serving your own kids, in your own community, and we just want to help you – encourage you in that (P8).

*School/community specific autonomy* also enabled communities themselves to decide how best to incorporate local Indigenous cultural traditions and values. One participant recalled a conversation about how IYMP was adapted to align with *“[their community’s] way of thinking about culture” (P2).* In their community, relationship building within IYMP included *“we’re sitting together, we’re eating together, and that’s one of the most important things that we do as [Indigenous] people is that every time we have a ceremony we sit and we eat together cause it’s about that sort of relationship building” (P2).* In another community, games were adapted to include the local Indigenous language.

#### Demonstrated administrative leadership

All participants agreed that support from administrators (i.e., school principal or centre director) was necessary to run IYMP. Participants identified administrators as leaders, and therefore, their support was considered vital to deliver the program. When administrators understood, valued, and prioritized IYMP, it supported implementation. Administrators also provided staff with space, resources, and time to run the program. One participant emphasized that the program ran well because *“[IYMP] always seemed to be like a key focus [for our administrator], like we had that time blocked off no matter what” (P9).* Administrators were also seen to play an important role in generating enthusiasm about the program, which made youth and staff more excited to get involved. As one participant explained:

We always have the principal onboard, and I can’t imagine what it would have been like to do those other projects without the principal on board because they bring in the teachers, they bring in the time, the energy. They guide the energies that are needed to make it happen (P5).

However, participants had differing views on administrators’ specific role. In some communities, participants felt that administrators needed to take an active role in the implementation of IYMP (i.e., recruiting, organizing, budgeting) whereas in other communities, participants felt that administrators needed to take a supportive role. Despite these differences, it was clear that active engagement from administrators could drive healthy changes within the school/community. For example, one participant illustrated how *demonstrated administrative leadership* in their school community made it possible to change the types of foods served not only for IYMP, but for all programs run out of their multi-use site. The participant explained:

We also realized that the [program] may not – they were trying, but they may not always have like the healthiest snacks … and what we ended up doing is we were talking to the [program] about it and [the administrator] decided that they would adopt the nutrition policy for the [site] (P2).

#### Higher-level support

Most participants valued provincial support to maintain and ripple the program. In the context of this study, *higher-level support* was described most often as provincial funding.

Interestingly, perceptions of *higher-level support* were notably different between YAHLs and coordinators. Thus their perspectives are described separately below. Among YAHLs, some believed that *higher-level support* was necessary to secure funding to maintain and ripple the program. Beyond financial support, participants felt as though securing funding meant that funders valued IYMP and prioritized the health and wellbeing of Indigenous youth. One YAHL stated that *higher-level support*, specifically funding, was very important for youth because:

They get to see that there is people higher up caring for us. Just like we are the ones caring for the ones, the grade fours. You know and it’s just the ripple effect, once everything starts going and it just keeps going (P13).

This was echoed by another YAHL when they stated: *“what are we doing to support the Indigenous people of this province, country? There’s a responsibility of, for all stakeholders, and on whatever level it is to participate in that” (P1).* Other YAHLs found it difficult to determine whether or not *higher-level support* was necessary because they felt as though their role within IYMP was focused on the day–to-day running of the program. A few recommended that we talk to coordinators and IYMP research partners to better understand the role of *higher-level support* within the context of IYMP.

While all coordinators agreed that provincial support was necessary to maintain and ripple the program, they also identified local leadership and support from Band Council, community advisory committees, and/or the school board as essential. One participant explained: *“[Band Council is] welcoming this program to the community and to their people. Well you need that support from the community, for sure” (P14).* In another community, local leadership included support from the school board, and a participant described the school board’s role as “*… very important … ‘cause it just wouldn’t have been allowed to happen without that” (P7).*

Finally, YAHLs and coordinators included the wider IYMP network of youth, community leaders, Elders, scientists, and knowledge users within their descriptions of *higher-level support*. One participant explained the role of the wider network as *“Mentorship is transformative, but the unique benefit [of IYMP] is that there is a wider network. [Communities] aren’t starting from scratch.” (P6).* Thus, a strength of IYMP is that communities are supported by the wider IYMP network, which includes direct support from coordinators in their region to take up and adapt the program. Similarly, coordinators described their role as building capacity within the community to implement IYMP. The wider IYMP network was also described as critical for providing learning opportunities, assisting with securing funding, and supporting research activities.

#### Dedicated champion(s) to engage school community

All participants agreed that the project requires at least one person from the community to organize IYMP and connect IYMP team members. More specifically, participants felt that the program would not have happened if there was not at least one person advocating for and organizing IYMP. As one participant explained: *“I think one person needs to be the lead and feel like they have ownership over it; and they’re going to making sure that it’s getting done every week, and that things aren’t falling through the cracks” (P6).* However, participants recognized that in order to promote the sustainability of IYMP, there needed to be multiple *dedicated champions*. One participant said: *“I think there would be probably need a team of people if you wanted to make it flourish in your school or your community” (P14).*

Unique to IYMP, participants identified the YAHL as an essential member of the team to plan and deliver the program. One participant described the role of the YAHL as “*planning activities with the students and helping to coordinate picking up the snack beforehand, and [they] played a huge role in the success of the program” (P9).* Beyond supporting implementation, YAHLs played an essential role in mentoring youth. One YAHL described their role as *“… trying to show them what mentorship can look like …*” *(P8).* Another indicated:

The little ones, they have little eyes, they already probably think about their lives' in high school and those are their role models. They interact with them in a way that is enjoyable, and the trust builds that way as well. And same for the mentors they look up to their YAHL. And that’s pretty cool to see. It’s like a full circle almost (P14).

Importantly, most YAHLs held other positions in the school or community organization where IYMP ran. This meant that they often had pre-existing relationships with students, teachers, and administrators and understood the context of the community. This was described as important because it meant that the YAHL had an understanding of how to work with youth and run programs in their community. This was exemplified by a participant when they stated:

Finding that right person you need to develop and implement the program and that wants to do it … Yeah because they’re the ones that create their program … I think it’s really important that you have that YAHL, that Young Adult Health Leader, who, you know, has those relationships with the high school mentors, is familiar with teachers and the system. That’s very valuable (P14).

Participants also believed that it was important for youth to have Indigenous YAHLs supporting them. As one participant shared, *“I am Métis myself, so [the primary researcher] thought that was like a good role for me to move into being a mentor for some of those high school students” (P9).* Another participant described how comforting it was to have support from Indigenous coordinators because *“… [they’re] there for support, and you know also being Indigenous being like is just really, really comforting” (P12).*

#### Community support

All participants indicated that support from the *school community* was essential to run the program, whereas only some participants felt that support from the *broader community* was essential for the successful delivery of the program. Participants indicated that support from the school/site where IYMP is delivered was needed to engage youth and gain the supports needed to run the program. One participant described how teachers’ active engagement in the program created *“… a very positive environment for the students” (P9).* Furthermore, participants highlighted the important contributions that their school community provided, which in turn made it possible to run the program. For example, one participant explained how it was easier to run a program when the school community was involved because *“[if] they have a dedicated bus driver, and they have access to a space, nutrition staff is just going to buy the food, then like logistics are a slam dunk” (P6)*.

In contrast, most participants indicated that relationships beyond the school/site could enhance the quality of the program, although these were not seen as essential. One participant expressed: *“teachers were very supportive…So we didn’t have a lot of interaction in the community with community members outside of the school environment” (P9).* Participants described hosting community events with parents to share information about IYMP. They also invited community members to plan activities with the students.

#### Quality and use of evidence

Participants valued the research component of IYMP as a way to gain support for the program, grow the program, and promote sustainability. One participant indicated: *“To keep it going in the long run, yes I think it’s important that the measurements are done in the beginning, and the end to show that there’s scientific proof that it is like helping us create stronger healthier children …” (P11).* Another explained:

[Communities] need to show families and council, and/or their board if they have an education board, um, they need to show them why this was worthwhile and so having those measurements throughout the year, and even like documenting it through photos and through videos; showing why this was worthwhile is really important for the sustainability of the program (P6).

The above quotes also illustrate how participants wanted to balance different types of evidence. However, participants tended to prioritize conversations, experiences, and stories. As one participant stated: “*If you hear it from the person who’s actually running it, or a high school mentor, then it becomes more alive … It’s all about bringing their voice. And they’re the best describers of [I] YMP in their community” (P14).*

Participants described using knowledge in the form of conversations, stories, and experience from YAHLs, coordinators, administrators, other school staff, and youth to adapt how the program was run. This was described by one participant as *“Trusting, empowering Indigenous ways of knowing” (P8).* Participants described using evidence throughout the process to enhance the quality of the program. In one community, sharing circles were encouraged as a way to make the program responsive to the interests of youth and mentors. One participant explained:

After every session with all the students, elementary, high school and the YAHLs, we would have a sharing circle at the end and just a reflection period, and we would hope – we would just talk, everyone would share what they thought went well, what they thought maybe we should improve on. Everyone sharing their feedback on how that session went (P9).

Participants also felt that another way to strengthen the program would be to enhance the sharing of knowledge/evidence between communities. One participant suggested that IYMP record how it is being implement in each community. Then, if a community has questions on how to run the program, they can reach out and ask:

… do you guys have any ideas of how to run a program … we have like 20 different communities that are trying the same exact thing, and they have – there’s 20 different ways that you could potentially run the program, so check out these (P8).

#### Learning opportunities

Participants agreed that *learning opportunities* supported the delivery and refinement of IYMP within their local school community. Regional, national, and training gatherings were seen as a strength of IYMP as they provided opportunities to bring together IYMP communities, provincial organizations, and academics who are passionate about this work. Participants emphasized the importance of face-to-face meetings to strengthen relationships and share knowledge between communities. One participant described their experience at a National Gathering as:

I think it was a great thing to have cause it kind of showed how communities across the country are running the programs, and every one kind of had different strategies and tips on how to facilitate the sessions, and just like great ideas of things you could do with the kids. And I don’t know, I thought they were – I thought they were very useful in just the fact that people were sharing ideas and things like that (P10).

Participants also felt strongly that *learning opportunities* were needed for youth as a way to support them in becoming better mentors. Specifically, participants agreed that *learning opportunities* for youth should focus on relationship building and include opportunities to get to know each other. As one participant explained: *“… we owe it to them to facilitate that, and so that is our goal, you know our first – our next meeting is just strictly building that community, the little community of mentors, and enjoying themselves, and having fun” (P1).* In addition, hands-on activities for youth were described as critical to develop the skills and confidence needed to lead the program. For example, one participant felt as though *learning* *opportunities* for youth were necessary *“… so that the kids feel confident planning it; identifying games and snacks to include; basic classroom management; basic team building; so that they as a group feel good together” (P6).* Thus, the condition was modified to focus wholistically on learning, as opposed to professional development opportunities.

### New Core condition

#### Rooted in relationships

YAHLs and coordinators emphasized how relationship building is a core component of IYMP, and therefore needed to be reflected within the essential conditions. This condition was developed, in part, based on participant feedback that the previously established contextual condition, *prior community connectivity*, was essential within the context of IYMP. One participant stated: *“I think its foundational and I think it’s what makes it successful”* (P5)*.* Similarly, another participant explained: “*it’s obviously important, so you feel like there’s trust … and people can feel safe” (P2).* Participants embraced and prioritized relationships by carving out space to be present, to listen and learn from one another, and to laugh together. The importance of relationships was reinforced by many participants at the end of the interview when they identified trusting relationships as one of the key factors needed to implement IYMP. This was emphasized by one participant who explained that other communities could successfully implement IYMP if *“[they have] that connectivity with the kids, high schooler [s]” (P13).*

Indeed, participants prioritized relationship building prior to and during implementation. Prior to implementation, participants felt that relationship building between administrators, local leadership, and champions was needed to develop trust. This was exemplified by one participant who mentioned: *“the relationships are the most important thing and that’s kind of – it’s very central to the program, so take the time to build those relationships first and then get the program started” (P7).* During implementation, participants felt that trusting relationships between youth, mentors, and YAHLs were the foundation for delivering IYMP. For example, one participant explained: “*I do think that, that’s why our program ran really smooth is that everyone started to feel so comfortable with each other and comfortable to share” (P9).* It was also important for those involved in the delivery of IYMP to develop trusting relationships and open communication to ensure that all the different aspects of IYMP, including planning, organization, and implementation ran smoothly. The importance of clear communication was emphasized by one participant who shared: *“I think the biggest thing for it to run was communication throughout the whole - like the higher up, all the way down” (P3).*

### Contextual conditions

#### Time

All participants believed that sufficient time was needed to successfully implement IYMP. Specifically, participants emphasized the need for dedicated time to plan, organize, and run the program. Participants found it easier to dedicate time to IYMP when it was seen as their role and responsibility rather than an additional commitment. Dedicated *time* to run the program was also seen as important for sustainability. One participant felt as though IYMP was sustainable in their community because IYMP fit well within their mandate to support youth programming. Thus, they had dedicated time to run the program. They explained:

I see a lot of programs where things start and one person starts it and tries to pass it off, and then it phases out. I really do believe that just under my mandate that, like as a team – with a teen mandate that I would, you know, definitely contribute to that sustainability (P3).

#### Funding and project support

Similarly, all participants agreed that funding made it easier to run the program. In order to include all three components of IYMP, communities needed space to run the program, equipment and supplies, transportation, and food. While some of these resources were provided in-kind, participants indicated that community-level funding made it possible to provide transportation and healthy snacks. As one participant stated: *“I think that’s definitely necessary, even just like paying for the food, and the buses, and all that kind of stuff I think it’s necessary to run the program in the way that we did run it” (P7).*

#### Readiness and understanding

Participants agreed that having a clear understanding of IYMP and why it is important made it easier to run the program. Participants felt that developing a clear understanding of the goals and purpose of the program, as well as their role within IYMP was important. Prior to implementation:

[communities need to] see how this can fit in [their] current schedule; why [it’s] worthwhile, like how it’ll help with student engagement; it’ll actually make your teachers feel less burnt out, because they’ll feel more connected to the students than they do now (P6).

In addition, communities felt that their understanding of the program and what was expected improved throughout implementation. As one participant explained:

The second time we ran it, we ran the program we were definitely a lot more clear about what the program was and what we were going to be doing, and what the goals of it were; and why it was great. And I think that made everyone feel a lot more comfortable and a lot less confused, as we went along (P10).

## Discussion

This study explored whether the essential conditions for taking a CSH approach resonated with participants’ experience implementing a peer-led health promoting intervention, IYMP in diverse Indigenous school communities in Canada. Overwhelmingly, participants agreed that the previously established essential conditions were necessary to implement IYMP within their community and/or the communities they work with. Although these conditions resonated with their experience, adaptations and the addition of a new condition, *rooted in relationships*, were needed to fit the context of IYMP. The materials developed from this study (see Fig. 1) represent a list of conditions identified by communities as necessary for program implementation and can be used to support the growth of IYMP. Our study adds to the literature by outlining what is needed in practice to implement a communal, relationship-based healthy living program for Indigenous youth. Previous studies have explored the implementation of community-based, health promotion programs for Indigenous youth [3, 5–7, 11]. However, the research presented here is unique in its inclusion of multiple communities and multiple stakeholders directly involved in the implementation of a best-practice intervention. These factors may promote the transferability of the reported findings to other healthy living programs for Indigenous youth, especially those within a school-based context.

To contextualize our findings, it is useful to explore how the essential conditions for implementing IYMP align with the current body of evidence. Participants in our study expressed that relationships were central to the implementation of IYMP. Therefore, a new condition, *rooted in relationships*, was created to reflect the importance of relationships prior to and during implementation. These results reaffirm findings from IYMP’s initial implementation study conducted in year 1, which identified building relationships as a key characteristic of implementation [39]. Outside of IYMP, the importance of relationship building within the context of community-based, participatory research is well established [16, 38, 40, 41]. In one study, First Nation communities and academics described “function[ing] well together” because their partnership was founded on trusting relationships [38]. Further, in a systematic review of school-based healthy living programs for Indigenous youth, it was suggested that the limited number of research studies in this area may be due, in part, to the need to develop long-standing relationships prior to implementation [1]. Based on these findings, when implementing healthy living programs for Indigenous youth, it is necessary to take time to build foundational relationships in each community prior to implementation. Relationships between youth, mentors, and YAHLs during implementation was also identified as essential. This is not surprising as a core component of IYMP is relationship building. Our findings reinforce the notion that centering programs on relationships as opposed to knowledge-based curriculum enhances implementation. Indeed, it has been proposed that centering programs on relationships enhances program relevance, an important attribute of program success [7, 16].

Within IYMP, the non-hierarchical, mentorship model closely aligns with a key Indigenous approach of reciprocal multi-age mentoring [16]. To our knowledge, this is the first study to identify the importance of relationship building for implementation within a communal, relationship-based healthy living program for Indigenous youth. Participants also highlighted the importance of relationship building within their descriptions of *dedicated champion(s) to engage school community* and *learning opportunities.* We learned how pre-existing relationships made it easier for YAHLs to deliver IYMP because they were not starting from scratch. Rather, those pre-existing relationships could serve as a foundation for the program. We also learned how relationship building between communities facilitated the sharing of knowledge, which enhanced implementation. Therefore, our data suggest that when rippling programs to multiple communities, it is important to identify key community partners with pre-existing relationships in each community. There also needs to be opportunities for relationship building across communities. Relationship building across communities has been shown to promote capacity building [3]. This may promote sustainability long-term as communities are supporting one another throughout the implementation process.

Closely tied to relationship building was youth enthusiasm, voice, and leadership. Participants reported that implementation was successful when youth were enthusiastic and actively participating as leaders. This aligns with the core tenants of health promotion to enable: “people and communities to take control over their health and its determinants” [42]. Previous research supports the notion that youth can act as change agents through peer to peer interactions because they are more likely to get involved, learn, and make changes when their peers are initiating it [12, 13, 43–45]. The data presented here reinforce these claims and extend them by revealing the role of relationships and mentorship in generating enthusiasm and engagement in healthy living programs for Indigenous youth. This study also highlights the importance of youth voice within the context of *quality and use of evidence.* Based on these findings, research in this area should prioritize youth voice to demonstrate the program and its impacts.

Another finding from our study that is well-supported in the literature is the need for *school/community-specific autonomy*. Other healthy living programs for Indigenous youth, including Sandy Lake Health and Diabetes Prevention Project, Kahnawà:ke Schools Diabetes Prevention Project, Right to Play, and Healthy Buddies have identified the importance of autonomy for implementation to ensure sufficient tailoring to the local context considering local traditions and values, and community resources [3, 7, 11]. This study adds to the literature by providing a description of what this could look like in practice when working with multiple communities. Participants described how those directly involved in IYMP were able to adapt how IYMP was delivered. This approach was described as successful because those involved had an understanding of their community and what would and would not work.

Participants also discussed the importance of *higher-level support.* Participants expressed how *higher-level support* might look different depending on what community you work with. Therefore, it was important to work with *dedicated champion(s)* in the community to identify local and provincial leaders to support implementation. An addition to the condition *higher-level support* was the inclusion of the wider IYMP network in supporting implementation. This network of youth, community leaders, Elders, scientists, and knowledge users provided support with implementation and evaluation, as well as *learning opportunities*. Taken together, IYMP appears to have been successful because it promotes autonomy and flexibility locally, while also developing a coordinated national network to connect and support communities. This aligns with the CSH approach [13]. One consideration then, is how IYMP can continue to support communities in a sustainable way. Therefore, more research is needed to understand how IYMP can transfer ownership from a community-academic partnership to community organizations to ensure that IYMP can continue in the absence of external funding. To better understand how this can be successfully achieved, participants in this study suggested that we speak with IYMP research partners, as they are actively engaged in this process. It would also be important to speak with the community organizations that may house IYMP moving forward.

Finally, participants in the study suggested a need for *learning opportunities* for youth. Re-wording this condition was important to broaden the scope of these experiences to include everyone who is involved in implementation, especially youth. *Learning opportunities* are seen as ways to build capacity and enable individuals to become their own health promotion experts [46]. Within the context of peer mentorship programs, learning opportunities for youth may be particularly beneficial as a way to enable youth to build confidence and expertise in health promotion at a young age.

### Strengths and limitations

This research was limited in some ways by the geographic scope of the IYMP program. For example, it was not possible for the lead author to meet with each participant in-person, so some interviews were conducted by phone. Conducting interviews by phone may have impacted the lead author’s ability to develop a rapport with participants and capture non-verbal cues. Another limitation of phone interviews was that the lead author was not able to take part in IYMP programing in that community. In communities where this was possible, it added context to the interviews and provided further information on what implementation looked like in each community. However, despite the limitations of phone interviewing, the data generated from these interviews enabled us to include a diversity of perspectives that otherwise would not have been captured.

A strength of the present research was the use of qualitative research methods. This approach provided a deeper understanding of how and why communities were able to successfully take up and implement IYMP in different settings. Indeed, traditional implementation science methods like program logs tend to oversimplify the implementation processes because they only identify what aspects of the intervention were implemented but not what conditions within the intervention process made it possible to implement or not implement the intervention [10]. Importantly, the latter is often more relevant for communities. Thus, this study not only fills an important gap in the literature, but it also fills a gap in practice. In addition, a novel aspect of this project was our ability to compare the experience of implementing IYMP from different perspectives and in different contexts. This is important because it enhances the transferability of our research findings and increases the likelihood that our findings are relevant and applicable to other communities and researchers. Finally, a strength of the present study was our ability to explore the process of implementing an effective, peer-led mentorship program for Indigenous youth. Indeed, this provides a scientific basis for understanding how this best-practice strategy can be rippled to new communities.

## Conclusions

In conclusion, our study supports the applicability of the essential conditions to the experience of implementing IYMP in diverse contexts. Participants agreed that the eight core conditions and four contextual conditions were necessary. They provided suggestions that resulted in adaptations to two core conditions: *youth led* and *learning opportunities*, as well as the development of a new condition: *rooted in relationships*. Participants also shared concrete examples of what the essential conditions look like in practice and how these conditions can be used to support the implementation of IYMP. These findings give direction for IYMP as they represent a list of conditions that have been identified as necessary for implementation by those directly involved in the delivery of IYMP. Moving forward, we recommend the use of these essential conditions to support on-going implementation and rippling of IYMP to new communities.

More broadly, findings from this study may provide guidance to researchers and communities implementing and/or rippling similar communal, relationship-based healthy living programs for Indigenous youth in school-settings. Therefore, we suggest these essential conditions be used when planning, implementing, and rippling healthy living programs for Indigenous youth, as they support the implementation of programs that are autonomous and fit the unique context and needs of communities.

## Supplementary Information

**Additional file 1.**

**Additional file 2.**

## Data Availability

In keeping with OCAP® principles, the data set generated during this study is not publicly available, as each community owns, controls, has access to and possesses their data. This respects that our communities are stewards of their own information. These data cannot be made available by the corresponding author on reasonable request as they are owned by the communities involved in this study. However, illustrative quotes are included throughout to provide supporting evidence.

## Abbreviations

CSH: Comprehensive School Health
IYMP: Indigenous Youth Mentorship Program
YAHL: Young Adult Health Leader
