# Supplementary material for: The essential conditions needed to implement the Indigenous Youth Mentorship Program: a focused ethnography
Source: BMC Public Health. 2022 Feb 2;22:213. doi: 10.1186/s12889-021-12412-1 (PMC8808991; doi:10.1186/s12889-021-12412-1)
Supplement: Supplementary file 1 — Additional file 1. [file 12889_2021_12412_MOESM1_ESM.pdf]

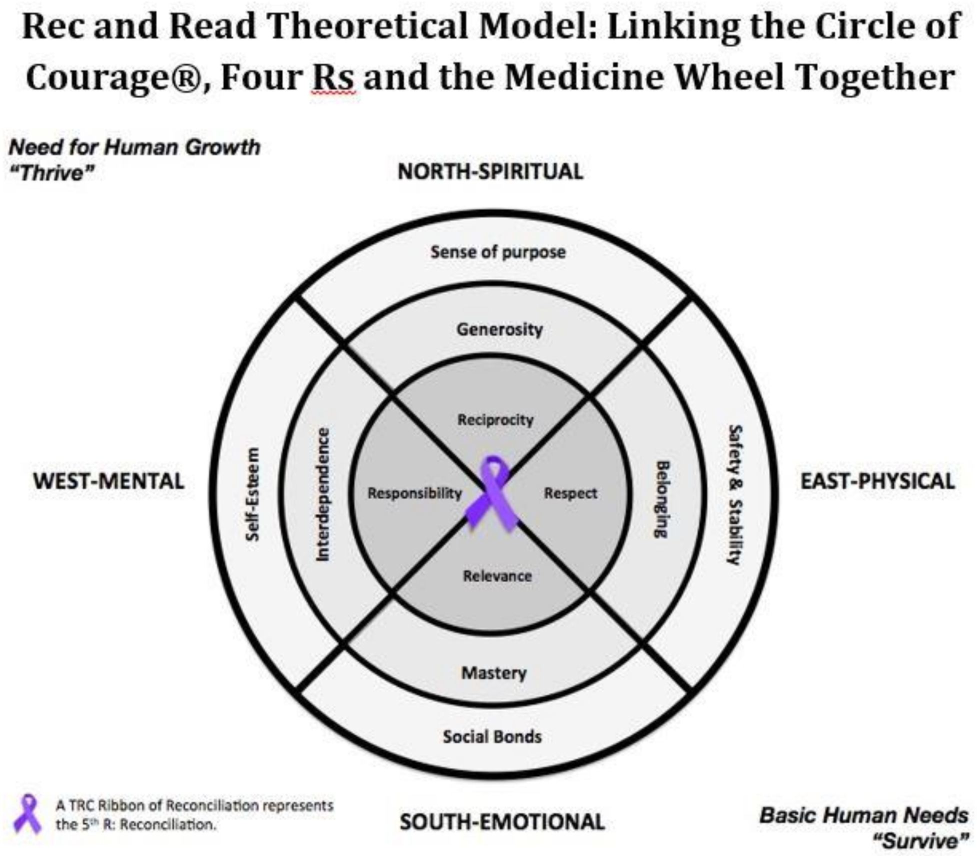

Supplementary Figure 1. The theoretical framework for IYMP was developed by Métis scholar and team member Dr. Heather McRae. It is based on the Indigenous medicine wheel and includes the teachings of two leading Indigenous scholars, Drs. Martin Brokenleg and Verna Kirkness. The inner circle depicts Dr. Kirkness' Four R's of learning that guide IYMP. Through its programming, IYMP strives to create an environment that fosters respect, relevance, reciprocity, and responsibility. The second ring describes Dr. Brokenleg's Circle of Courage and the four key elements that mentors are asked to bring to IYMP. IYMP builds on the strengths, enthusiasm, and experiences of youth to create opportunities for youth to experience belonging, mastery, independence, and generosity. Finally, the outer ring describes the well-being and health-related outcomes that emerge from the inner circles and that IYMP provides: Safety and Stability, Social Bonds, Self-Esteem, and Sense of Purpose.
