# Supplementary material for: The essential conditions needed to implement the Indigenous Youth Mentorship Program: a focused ethnography
Source: BMC Public Health. 2022 Feb 2;22:213. doi: 10.1186/s12889-021-12412-1 (PMC8808991; doi:10.1186/s12889-021-12412-1)
Supplement: Supplementary file 2 — Additional file 2. [file 12889_2021_12412_MOESM2_ESM.docx]

**IYMP National Team**

Western Door
Arcand, Roy; Esau, Keri; Ferguson, Leah; Kootenay, Jody; Lopresti, Sabrina; McHugh, Tara-Leigh; Sobierajski, Frances; Storey, Kate E; Torrance, Brian; Torres Ruiz, Maria Fernanda; Willows, Noreen D

Central Door

Beardy, Tara-Lee; Carlson, Barbara; Enosse, Lawrence; Fewchuk, Rick; Halas, Joannie M;

Johnson, Jay; McGavock, Jonathan M; McIvor, Connie; McRae, Heathe; Robinson, Jack; Roulette, Rene; Wabano, Mary-Jo; Wood, Eric; Wood, Larry

Eastern Door

Baillie, Colin; Lévesque, Lucie; McComber, Alex M; Poulette, Addy; Young, Nancy L
